# Repair of deep deltoid ligament ruptures near the medial malleolar attachment or midsubstance rupture by using suture anchors into the talus combined with the transosseous suture in the medial malleolar

**DOI:** 10.3389/fsurg.2023.1287427

**Published:** 2024-01-05

**Authors:** Wei Liang, Mingping Zhou, Zhongting Jiang, Xuanyu Mao, Xiang Zhou, Fei Wang

**Affiliations:** ^1^ Department of Orthopaedics, Lishui People's Hospital, The Sixth Affiliated Hospital of Wenzhou Medical University, The First Affiliated Hospital of Lishui University, Lishui, Zhejiang, China; ^2^ Department of Orthopaedics, Longquan People’s Hospital, Longquan, Zhejiang, China

**Keywords:** deltoid ligament rupture, suture anchor, transosseous suture, medial malleolar, talus

## Abstract

**Purpose:**

For deep deltoid ligament ruptures near the medial malleolar attachment, anchors were usually placed at the posterior colliculus and intercollicular groove. However, this procedure usually requires a prolonged surgical incision to fully expose the deep deltoid ligament, causing more trauma. In order to reduce surgical trauma, we explored the treatment outcomes of suture anchor into the talus combined with transosseous suture in the medial malleolar for the treatment of deep deltoid ligament ruptures near the medial malleolar attachment or midsubstance rupture.

**Patients and methods:**

This is a retrospective study of patients who received suture anchor into the talus combined with transosseous suture in the medial malleolar for repairing deltoid ligament ruptures near the medial malleolar attachment or midsubstance rupture. The outcome measures include the American Orthopaedic Foot and Ankle Society (AOFAS) ankle-hindfoot score, visual analogue scale (VAS), and the active range of motion (ROM) of the ankle at the final follow-up visit after surgery. Medial malleolus gap was evaluated by radiographic examination.

**Results:**

This study included 64 patients. The mean follow-up time was 36.3 ± 15.2 months. There were 43 patients with injuries on the medial malleolar side, and 21 cases on the midsubstance. The average AOFAS and VAS were 87.5 ± 4.9 and 0.7 ± 0.5, respectively. No significance in medial malleolus gap between the contralateral side and affected side was observed.

**Conclusion:**

For deltoid ligament ruptures near the medial malleolar attachment or midsubstance rupture, suture anchor into the talus combined with transosseous suture in the medial malleolar yields good clinical effect and outcome, is an optimal management of ankle syndesmosis injuries.

## Introduction

The deltoid ligament origins at the medial malleolus and fans out to the medial side of the talus, calcaneus and navicular bones, including superficial and deep ligaments (1). It plays an important role in stabilizing the medial side of the ankle joint (2–4). The medial malleolar attachment is the most common site of deltoid ligament rupture (5, 6). For ankle fractures complicated with deltoid ligament ruptures, surgical intervention for ruptured deltoid ligament remains controversial (7, 8). Failure to repair the deltoid ligament may be complicated by ankle joint instability and loss of ankle function, leading to traumatic arthritis (9). Thus, repair of deltoid ligament ruptures is usually performed along with internal fixation of the ankle fracture.

Suture anchor was usually inserted according to the rupture site of the deep deltoid ligament. If the deltoid ligament was ruptured near the medial malleolar attachment, the suture anchor was usually inserted into the medial malleolus. If the deltoid ligament was ruptured near the talar attachment, the suture anchor was usually inserted into the talus. For the case of midsubstance rupture of deltoid ligament, the deltoid ligament rupture was directly sutured. Previous studies showed that suture anchors into the talus had some unique advantages. The talar insertion area of the deltoid ligament is much broader than its origin in the medial malleolus (1), which is helpful to avoid the iatrogenic fracture of the medial malleolus when the suture anchor was inserted. Additionally, this procedure usually does not require a prolonged surgical incision and cause less trauma.

To date, there is no study reporting suture anchor into the talus for treating deltoid ligament ruptures near the medial malleolar attachment or midsubstance rupture. Thus, the study first evaluates the clinical outcomes of suture anchor into the talus combined with transosseous suture in the medial malleolar for treating such deltoid ligament ruptures and promote its clinical application.

## Material and methods

### Study population

From Dec 2020 to Jun 2021, 64 patients with deltoid ligament ruptures in ankle fractures operated in our hospital were retrospectively analyzed. Deltoid ligament integrity was assessed by the presence of radiographic ankle dislocation and medial clear space wider than 4 mm, and intraoperative exploration. Our study was approved by the Ethics Committee of our hospital and was in accordance with the Helsinki Declaration. All patients gave informed consent and signed informed consent.

Inclusion criteria of this study were (1) deltoid ligament full-layer ruptures in ankle fractures, which were determined by both preoperative MRI and intraoperative exploration; (2) deltoid ligament ruptures near the medial malleolar attachment or midsubstance rupture, which were determined by both preoperative MRI and intraoperative exploration; (3) those who were treated by suture anchor into the talus combined with transosseous suture in the medial malleolar. Exclusion criteria of this study were (1) Pathological fractures and open fractures (2) Those who cannot tolerate surgery (3) Follow-up data were incomplete and the follow-up time was less than 12 months.

### Surgical techniques

The surgical procedure of deltoid ligament repair was as follows:

(1) Vascular clamp was used to clear the synovial tissue outside the deltoid ligament, clearly reveal the broken end of the deltoid ligament, and identify the anterior and posterior edges of the ligament.
(2) Two suture anchors were inserted at the talus, which were located at the anterior and posterior edges of the deep deltoid ligament.
(3) A horizontal line with the articular surface of the lower tibia was drawn 1 cm above the tip of the medial anterior colliculus. At the intersection of the line with the middle and posterior 1/3 of the anterior colliculus and the anterior and middle 1/3 of the posterior colliculus, two holes were drilled with 2.0 mm Kirkler's needle at the talus attachment. Transosseous suture can provide stable repair.
(4) We passed the tail line of the suture anchors through the two broken ends of the ligament (midsubstance rupture) or the whole process (medial malleolar attachment rupture), and then through the reserved holes in the medial malleolar. We tightened the tail line of the suture anchors and tied the knot after the talus—medial ankle relationship was restored with the reduction forceps. The superficial deltoid ligament can be repaired directly or by the suture anchor inserted at the anterior medial malleolar according to the deltoid ligament rupture site. The broken end of the ligament was investigated to confirm that the end-to-end involution was good at the broken end.
(5) The excess part of the tail line of the suture anchors was used to suture the superficial layer of the deltoid ligament and the joint capsule.
(6) After fractures were anatomically reduced and fixed, the ankle joint varsion external rotation stress test with x-ray was performed to observe the stability of the medial malleolus and the lower tibiofibular union. Static or dynamic fixation of the lower tibiofibular union was performed if necessary.

### Assessments

All patients were followed up for at least 12 months. Clinical characteristics, cause of injury, and complications were recorded. The time to fracture healing and the time to full bear weight were documented during the follow-up. The clinical outcomes include the American Orthopaedic Foot and Ankle Society (AOFAS) ankle-hindfoot score, visual analogue scale (VAS), and the active range of motion (ROM) of the ankle. In addition, medial malleolus gap was evaluated through x-ray.

### Statistical methods

All statistical analyses were performed with SPSS 23.0 (IBM, Armonk, NY). Quantitative variables were presented in the form of mean ± standard deviation and categorical variables were shown in the form of frequency (%). A paired *t*-test was used for comparing medial malleolus gap. Variables with two-tailed *p* < 0.05 were considered statistically significant.

## Results

### Patient characteristics

A total of 64 patients were included in this study. Of the 64 patients, 43 were male (67.2%) and 21 were female (32.8%), with a mean age of 43.6 ± 8.8 years. According to deltoid ligament rupture site, 43 patients had injuries on the medial malleolar side, and 21 cases on the midsubstance. The causes of injury were falling from height (*n* = 6), twist (*n* = 33), and traffic accident (*n* = 23). All patients were followed up. The average postoperative follow-up duration was 36.3 ± 15.2 months (Table 1).

**Table 1:** Patient demographics.

Variables	All (*n* = 64)
Mean age, years	43.6 ± 8.8
Gender, *n* (%)	
Male	43 (67.2%)
Female	21 (32.8%)
Deltoid ligament rupture site	
Medial malleolar side	43 (41.5%)
Midsubstance	21 (58.5%)
Follow-up, months	36.3 ± 15.2
Cause of injury, *n* (%)	
Fall from height	6 (9.4%)
Twist	33 (51.6%)
Traffic accidents	23 (35.9%)

Mean ± standard deviations for the two groups.

### Clinical outcomes

The mean full weight-bearing time was 3.1 ± 0.3 months. The mean AOFAS was 87.5 ± 4.9 and the average VAS was 0.7 ± 0.5. Additionally, the average ankle ROM of all patients was 60.1 ± 3.5 degrees. According to the postoperative radiological examination, there was no significance in medial malleolus gap between the contralateral side and affected side (*p* > 0.05) (Table 2).

**Table 2:** Clinical and radiographic outcomes.

Variables	All (*n* = 64)
Full weight-bearing time, month	3.1 ± 0.3
AOFAS	87.5 ± 4.9
VAS	0.7 ± 0.5
Ankle ROM, degree	60.1 ± 3.5
Contralateral medial malleolus gap, mm	3.2 ± 0.2
Affected side medial malleolus gap, mm	3.2 ± 0.2

Mean ± standard deviations for the two groups.

Wounds healed well in all patients. No neural damage, and deltoid re-rupture occurred. No infection and fracture nonunion were observed during the follow-up.

**Figure 1:**
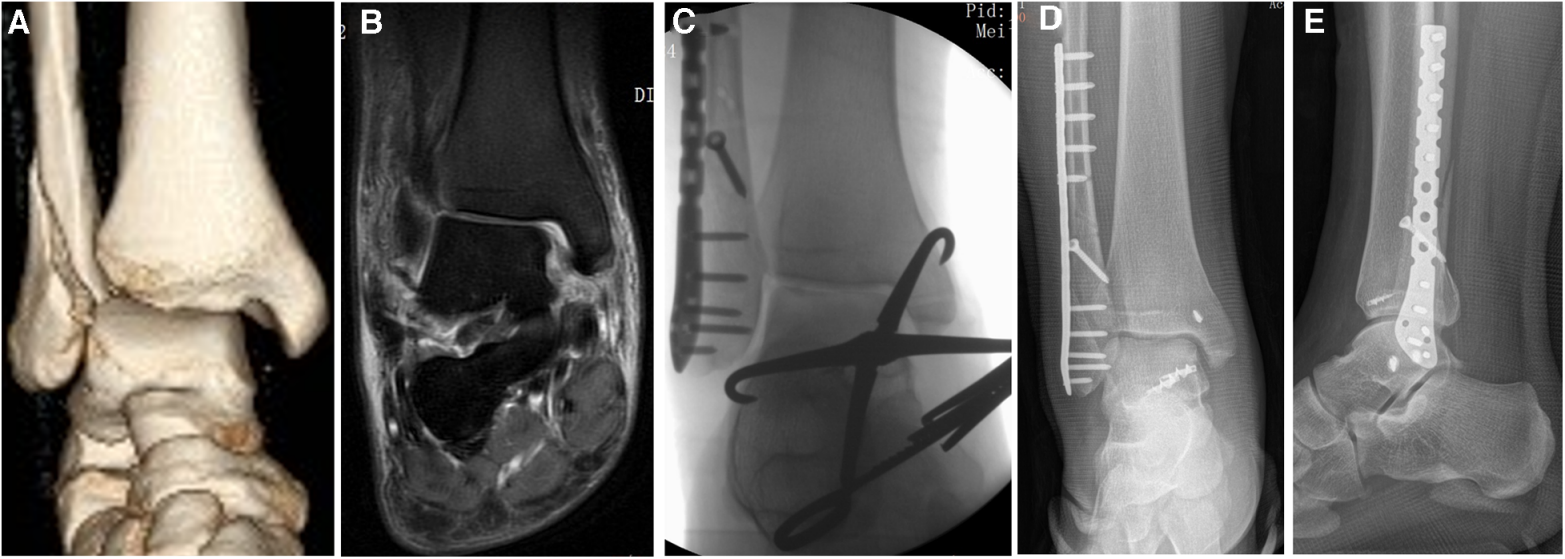
A case of repairment for deep deltoid ligament rupture near the medial malleolar attachment by using suture anchors into the talus combined with transosseous suture in the medial malleolar. (**A**) Preoperative 3D reconstruction image showed increased medial malleolar space and ankle fracture. (**B**) Preoperative coronal MRI showed that the ruptured deltoid ligament. Combined with intraoperative exploration, the deltoid ligament was ruptured near the medial malleolar attachment. (**C**) Intraoperative anterior–posterior x-ray image showed the fixation of the talus—medial ankle relationship with reduction forceps. (**D**) Postoperative anterior–posterior x-ray image. The deep deltoid ligament was repaired by two suture anchors inserted at the talus. The superficial deltoid ligament was repaired by one suture anchor inserted at the medial malleolar. (**E**) Postoperative lateral x-ray image.

Figure 1 showed one typical case of repairment for deep deltoid ligament rupture near the medial malleolar attachment by using suture anchors into the talus combined with transosseous suture in the medial malleolar.

## Discussion

Deltoid ligament injuries in ankle fractures are very common in clinical orthopaedics. Hintermann et al. (10) used arthroscopy to confirm that 40% of cases with ankle fractures were accompanied by deltoid ligament injuries, and the probability of complete deltoid ligament ruptures was 22.2%. However, there is no consensus on deltoid ligament repair in ankle fractures (4, 11, 12). Although recent studies showed no differences in long term functional outcomes when the deltoid ligament was repaired (13), the deltoid ligament repair may lead to better ankle stability (11, 14). Thus, more and more researchers believe that surgical repair of deltoid ligament rupture is the first choice to maintain the stability of ankle joint and prevent the occurrence of valgus and extransion of talus and long-term traumatic arthritis (15–17). In the present study, good functional outcomes were achieved and technique safety was observed based on the results of AOFAS, VAS, and ankle ROM. No significance in medial malleolus gap between the contralateral side and affected side indicated that there was no medial instability of the ankle joint after surgery. Additionally, no complication was observed in our study.

Deltoid ligaments are usually repaired according to the different positions of ruptures. During the operation, we found that after the reduction of the medial malleolar space, the direct end-to-end suture of the midsubstance rupture was more difficult. Iatrogenic fracture of the medial ankle would occur when the anchor was treated at the medial ankle. Therefore, for deltoid ligament ruptures near the medial malleolar attachment or midsubstance rupture, we adopted the same method as that of deltoid ligament ruptures near the talar attachment. When the posterior and lateral structures of the ankle joint were not fixed and the medial malleolar space was not reduced, each suture anchor was inserted at the anterior and posterior edges of the deltoid ligament insertion point near the talus. After the reduction and fixation of the external and posterior ankle fractures were completed, the internal ankle space was restored with the reduction forceps, and then the tail lines of the suture anchors were tightened to tie the knot. At the same time, the anastomosis of the severed ligaments could be observed. This could avoid the difficulty of ligament suture in the narrow space. To prevent bone dissection during the transosseous suture at the medial malleolar, we covered the drills with the surrounding soft tissue, and then tied the knot of the tail line over the soft tissue.

Chen et al. (18) found that deep deltoid ligament repair in treating supination-external rotation fracture displayed enhanced functional outcome, with an average AOFAS of 92.8 and a VAS of 0.79 at 12 months. For the ligament rupture at the endpoint of the medial malleolus, they placed two anchors at the posterior colliculus and intercollicular groove. However, this method usually requires a prolonged surgical incision to fully expose the deep deltoid ligament, with a corresponding increase in injury. Nacime et al. (19) repaired the deltoid ligament rupture arthroscopically and achieved an average AOFAS of 93.5 and a VAS of 0.75. They passed sutures using a suture passer, respecting the safe zone, on the area between the posterior tibial tendon and the saphenous vein (16–25 mm) (20). The average AOFAS and VAS in our study were 87.5 ± 4.9 and 0.7 ± 0.5, which was similar with their results. Overall, the results of our study suggest that suture anchor into the talus combined with transosseous suture in the medial malleolar for the treatment of deltoid ligament ruptures near the medial malleolar attachment or midsubstance rupture can achieve good clinical efficacy, which is worthy of clinical promotion and application.

There are several limitations in this study. First, the retrospective nature of this study can generate bias. Second, this study was limited by the relatively small sample. Relevant clinical studies with larger sample sizes can be carried out in the future. Additionally, larger multi-center, prospective, randomized studies need to be carried out.

## Conclusion

Suture anchors into the talus combined with transosseous suture in the medial malleolar is a favorable treatment strategy for the deltoid ligament ruptures near the medial malleolar attachment or midsubstance rupture.

## Data Availability

The raw data supporting the conclusions of this article will be made available by the authors, without undue reservation.

## References

[1] Campbell KJ Michalski MP Wilson KJ Goldsmith MT Wijdicks CA LaPrade RF The ligament anatomy of the deltoid complex of the ankle: a qualitative and quantitative anatomical study. J Bone Joint Surg Am. (2014) 96(8):e62. eng. 10.2106/jbjs.M.00870 24740670

[2] Loozen L Veljkovic A Younger A. Deltoid ligament injury and repair. J Orthop Surg (Hong Kong). (2023) 31(2):10225536231182345. eng. 10.1177/10225536231182345 37449811

[3] Stufkens SA van den Bekerom MP Knupp M Hintermann B van Dijk CN. The diagnosis and treatment of deltoid ligament lesions in supination-external rotation ankle fractures: a review. Strategies Trauma Limb Reconstr. (2012) 7(2):73–85. eng. 10.1007/s11751-012-0140-9 22767333 PMC3535131

[4] Güvercin Y Abdioğlu AA Dizdar A Yaylacı EU Yaylacı M. Suture button fixation method used in the treatment of syndesmosis injury: a biomechanical analysis of the effect of the placement of the button on the distal tibiofibular joint in the mid-stance phase with finite elements method. Injury. (2022) 53(7):2437–45. eng. 10.1016/j.injury.2022.05.037 35641331

[5] Crim J Longenecker LG. MRI And surgical findings in deltoid ligament tears. AJR Am J Roentgenol. (2015) 204(1):W63–9. eng. 10.2214/ajr.13.11702 25539277

[6] Woo SH Bae SY Chung HJ. Short-term results of a ruptured deltoid ligament repair during an acute ankle fracture fixation. Foot Ankle Int. (2018) 39(1):35–45. eng. 10.1177/1071100717732383 29078057

[7] Park YH Jang KS Yeo ED Choi GW Kim HJ. Comparison of outcome of deltoid ligament repair according to location of suture anchors in rotational ankle fracture. Foot Ankle Int. (2021) 42(1):62–8. eng. 10.1177/1071100720952053 32951566

[8] Wang J Stride D Horner NS Petrisor B Johal H Khan M The role of deltoid ligament repair in ankle fractures with syndesmotic instability: a systematic review. J Foot Ankle Surg. (2021) 60(1):132–9. eng. 10.1053/j.jfas.2020.02.014 33218869

[9] Guo W Lin W Chen W Pan Y Zhuang R. Comparison of deltoid ligament repair and non-repair in acute ankle fracture: a meta-analysis of comparative studies. PLoS One. (2021) 16(11):e0258785. eng. 10.1371/journal.pone.0258785 34767584 PMC8589189

[10] Hintermann B Regazzoni P Lampert C Stutz G Gächter A. Arthroscopic findings in acute fractures of the ankle. J Bone Joint Surg Br. (2000) 82(3):345–51. eng. 10.1302/0301-620x.82b3.10064 10813167

[11] Salameh M Alhammoud A Alkhatib N Attia AK Mekhaimar MM D'Hooghe P Outcome of primary deltoid ligament repair in acute ankle fractures: a meta-analysis of comparative studies. Int Orthop. (2020) 44(2):341–7. eng. 10.1007/s00264-019-04416-9 31776609 PMC6968990

[12] Güvercin Y Yaylacı M. Biomechanical investigation of the effects of various treatment options on the talus in supination external rotation type 4 ankle injuries with ruptured deltoid ligament: finite element analysis. Sakarya Med J. (2023) 13(1):62–9. 10.31832/smj.1220996

[13] Bastias GF Filippi J. Acute deltoid ligament repair in ankle fractures. Foot Ankle Clin. (2020) 25(4):597–612. eng. 10.1016/j.fcl.2020.08.009 33543718

[14] Yang X Zeng J Yang W Dela Rosa RD Jiang Z. A meta-analysis of deltoid ligament on ankle joint fracture combining deltoid ligament injury. Front Surg. (2023) 10:976181. eng. 10.3389/fsurg.2023.976181 37051572 PMC10083234

[15] Liao J Zhang J Ni W Luo G. A retrospective study of deltoid ligament repair versus syndesmotic fixation in lateral malleolus fracture combined with both deltoid ligament injury and inferior tibiofibular syndesmotic disruption. Front Surg. (2022) 9:912024. eng. 10.3389/fsurg.2022.912024 36386501 PMC9645235

[16] Hardy MA Connors JC Zulauf EE Coyer MA. Acute deltoid ligament repair in ankle fractures: five-year follow-up. Clin Podiatr Med Surg. (2020) 37(2):295–304. eng. 10.1016/j.cpm.2019.12.004 32146985

[17] Li H Xue X Tong J Li H Hua Y. Deltoid ligament (DL) repair produced better results than DL nonrepair for the treatment for rotational ankle instability. Knee Surg Sports Traumatol Arthrosc. (2023) 31(5):2015–22. eng. 10.1007/s00167-022-07169-y 36175528

[18] Chen H Yang D Li Z Niu J Wang P Li Q The importance of the deep deltoid ligament repair in treating supination-external rotation stage IV ankle fracture: a comparative retrospective cohort study. Biomed Res Int. (2020) 2020:2043015. eng. 10.1155/2020/2043015 33313312 PMC7719498

[19] Mansur NS B Raduan FC Lemos A Baumfeld DS Sanchez GT do Prado MP Deltoid ligament arthroscopic repair in ankle fractures: case series. Injury. (2021) 52(10):3156–60. eng. 10.1016/j.injury.2021.06.020 34247766

[20] Acevedo JI Kreulen C Cedeno AA Baumfeld D Nery C Mangone PG. Technique for arthroscopic deltoid ligament repair with description of safe zones. Foot Ankle Int. (2020) 41(5):605–11. eng. 10.1177/1071100720909138 32100553

